# Stem Cell-Based Dental Tissue Engineering

**DOI:** 10.1100/tsw.2010.81

**Published:** 2010-05-18

**Authors:** Petar Zivkovic, Vladimir Petrovic, Stevo Najman, Vladisav Stefanovic

**Affiliations:** University School of Medicine, 18000 Nis, Serbia

**Keywords:** tooth development, stem cells, tooth engineering, dentin-pulp complex, periodontium

## Abstract

The development of biological and biomaterial sciences profiled tissue engineering as a new and powerful tool for biological replacement of organs. The combination of stem cells and suitable scaffolds is widely used in experiments today, in order to achieve partial or whole organ regeneration. This review focuses on the use of tissue engineering strategies in tooth regeneration, using stem cells and stem cells/scaffold constructs. Although whole tooth regeneration is still not possible, there are promising results. However, to achieve this goal, it is important to understand and further explore the mechanisms underlying tooth development. Only then will we be able to mimic the natural processes with the use of stem cells and tissue engineering techniques.

## INTRODUCTION

The loss of teeth is one of the most common functional and esthetic defects in society today. The increased number of cases of periodontal diseases among the older population, as well as caries and traumas, are the leading causes of tooth loss[1]. Tooth transplantation and dental implants are the most used therapeutic approaches in restorative dentistry today in order to preserve the masticatory function of patients. However, complete restoration therapy to compensate for complete tooth loss has not been achieved[1].

The development of biological sciences, the profiling of tissue engineering, and the discovery of stem cells have offered new possible solutions in the therapy of tooth loss. Although the ideal solution to the problem would be the creation of the whole tooth by using the patient's own stem cells, we are still far from that goal because the biological processes underlying tooth development and differentiation are still not completely clear.

## TIMETABLE OF TOOTH DEVELOPMENT: A POSSIBLE TEMPLATE FOR TOOTH REGENERATION

The tooth develops through complex reciprocial interactions between the ectodermal-derived oral epithelium and neural crest–derived ectomesenchyme[2]. Considering that there is only a slight difference in the gene expression between murine and human tooth development, mice are commonly used as a model for tooth development[1]. The mediators in these processes are signal molecules belonging mostly to the four conservative families: tumor growth factor beta (TGF-β), wingless integrated (Wnt), fibroblast growth factor (FGF), and hedgehog[3].

The first event in tooth development is the thickening of the oral epithelium and its proliferation into the underlying mesenchyme, in the form of the cellular cord called dental lamina[2]. The oral epithelium possesses the inductive capability for odontogenesis, which leads to the conditionning of the underlying mesenchyme. Around E10 (embryonic day 10) of mouse tooth development, Bmp4 and Fgf8 are expressed in the dental epithelium. These two molecules are crucial for tooth type specification and for restriction of the tooth-forming sites[4,5,6,7].

At the bud stage, the dental mesenchyme acquires odontogenic potential. BMP-4 is belived to be the main component of the odontogenic potential[8].

Cap stage occurs at E14.5 when epithelial components undergo specific folding, which is accompanied by the formation of the enamel knot under the influence of BMP-4. The enamel knot represents the signaling center that determines tooth morphogenesis, apoptosis, and definite tooth morphology[9,10]. By the late cap stage, enamel knots disappear by apoptosis[11].

At the cap stage on E15, the mouse embryo mesenchyme undergoes differentiation into two distinct types of cells: dental papilla cells and dental follicle cells[12]. The bell stage occurs around E17. At the bell stage, the tooth germ consists of the enamel organ, dental papilla, and dental follicle. At this time, dental papilla cells differentiate into the cells of odontogenic lineage (odontoblasts and dental pulp stem cells)[13].

## ROOT AND PERIODONTIUM FORMATION

The dental follicle is the transient structure during tooth development that is formed from the cranial neural crest–derived dental mesenchyme on E15. The dental follicle has three major types of cells: cementoblasts, osteoblasts, and fibroblasts.

Formation of the periodontium happens simultaneously with the root formation. After the crown formation, the apical mesenchyme of the dental papilla continues to proliferate to form the developing periodontium, while, at the same time, cells of the cervical loop proliferate and form the bilayer Hertwig's epithelial root sheath and produce the basement membrane that contains chemotactic proteins that would induce cementoblast differentiation[14]. One of these proteins is the cementum attachment protein (CAP) whose role is in recruiting putative cementoblast precursors from the dental ectomesenchyme[15].

Transcription factors associated with root development include two members of the homeobox family of transcription factors: Dlx2 and Dlx3[16]. The Nfi-c transcription factor also seems to play an important role in root development. Nfi-c knockout mice appear normal, except that they exfoliate their teeth shortly after eruption. These mice show a lack of roots of both the mandibular and maxillary teeth, and therefore their teeth have no bone attachment[17].

Periodontal ligament (PDL) formation occurs simultaneously with the cementogenesis. PDL progenitor cells from the dental follicle differentiate into fibroblasts that start to secrete collagen fibers that become organized into tendinous-like tissue that connects the tooth with the alveolar bone[18,19].

PDL-associated protein PLAP-1/asporin, which belongs to the novel SLRP family, is specifically expressed in the dental follicle and adult PDL. This protein directly interacts with BMP-2, thus preventing the mineralization of PDL[20]. Another protein, periostin, was reported in the dental follicle. This protein becomes restricted in postnatal PDL during root formation[21]. It was shown that mice deficient in periostin have disorganized PDL and alveolar bone resorption[22]. Also, the role for development of PDL was suggested for another protein, F-spondin[23].

## ADULT STEM CELLS FOR TOOTH ENGINEERING

A stem cell is self-renewable and capable of differentiating into at least two distinctive cell types[24]. The ability for self-renewal means that undifferentiated daughter cells are precisely the same as the mother cell and can further replicate many generations without losing their original characteristics[25]. Multilineage differentiation refers to the capacity of a single population of stem cells to differentiate into at least two distinctively different cell types[24,25].

Stem cells used in dentistry may be divided into two major categories: embryonic stem cells and somatic (adult) stem cells. Somatic stem cells are limited in their potential for differentiation. There are opinions that somatic (adult) stem cells are a better option for dentistry because they are easily accessible and their use does not bring up ethical concerns[26].

Nonhematopoietic, bone marrow–derived, mesenchymal stem cells (BMDSC) can be isolated from single-cell suspensions from bone marrow aspirates[27]. First described in bone marrow, BMDSC have been extensively characterized *in vitro* by the expression of markers, such as STRO-1 (a cell surface antigen used to identify osteogenic precursors in bone marrow), CD146 (a pericyte marker), and CD44 (a mesenchymal stem cell marker)[27,28]. BMDSC possess a high self-renewal capacity and the potential to differentiate into mesodermal lineages, thus forming cartilage, bone, adipose tissue, skeletal muscle, and the stroma of connective tissues[29]. Furthermore, these cells have the capacity of differentiating into hepatocytes and renal, cardiac, and neural cells[30]. In many studies, dental cell therapies have been discussed by combining nondental mesenchymal stem cells and dental stem cells[31,32,33,34]. The positive effect of enamel matrix proteins on porcine BMDSC differentiation into cementoblasts was demonstrated[35]. It was shown, for the first time, that bone marrow–derived cells can be reprogrammed to give rise to ameloblast-like cells, offering novel possibilities for regenerative dentistry[36].

Dentin pulp stem cells (DPSC) were first isolated and identified in 2000 by Gronthos and coworkers[37]**.** Dental stem cells are located in the various regions of the dental pulp and express the stem cell marker STRO-1, vascular cell marker CD146, and pericyte antigen 3G5[38]. DPSC show a 30% higher proliferation rate than BMDSC under the same culture conditions, probably because of strong expression of cyclin-dependent kinase 6, a cell cycle activator[39]. In contrast to BMDSC, DPSC were found to be able to differentiate into odontoblast-like cells. DPSC were also found to differentiate into adipocytes, osteoblasts, smooth and skeletal muscle cells, chondroblasts, and neural-like cells under chemically defined culture conditions[40]. DPSC can be cryopreserved and retain their multipotential differentiation ability[41].

Stem cells from exfoliated deciduous teeth (SHED) may be isolated from the dental pulp of exfoliated incisors. SHED exhibit a high plasticity and they can differentiate into neurons, adipocytes, osteoblasts, and odontoblasts[38]. They induce bone formation and produce dentin under *in vivo* conditions. These cells are also able to survive and migrate in the murine brain after transplantation into immunocompromised animals[42]. Compared to DPSC, SHED show a higher proliferation rate and probably represent a population of more immature multipotent stem cells than DPSC.

The PDL was also found to contain STRO-1–positive cells. PDL stem cells (PDLSC) can adopt adipogenic, osteogenic, and chondrogenic phenotypes *in vitro*[43]. It is evident that the PDL itself contains progenitors, which can be activated to self-renew and regenerate PDL, cementum, and alveolar bone[44].

Dental follicle precursor cells (DFPC) are plastic, adherent, colony-forming cells, and they can differentiate into osteoblast-like cells under *in vitro* conditions. They have been isolated from the follicle of human third molars. Like PDLSC, DFPC can also differentiate and produce clusters of mineralized tissue[45,46,47]. DFPC express the mesenchymal stem cell marker STRO-1 and have multipotential mesenchymal precursor cell properties[45]. DFPC can differentiate toward multiple mesenchymal-derived cell types, such as cementoblasts, chondrocytes, and adipocytes.

Stem cells from dental apical papilla (SCAP) are easily accessible since they can be isolated from human third molars. SCAP have the potential to differentiate into odontoblasts[48]. SCAP exhibit a higher proliferative rate and appear to be more effective than PDLSC for tooth formation. SCAP have a greater capacity for dentin regeneration than DPSC because the dental papilla contains a higher number of adult stem cells compared to the mature dental pulp. A combination of SCAP and PDLSC may induce the formation of a dental connective tissue, the attachment of an artificial tooth crown in the alveolar bone[48].

## STEM CELLS AND TISSUE ENGINEERING

Tissue engineering, according to the definition from the National Institute of Health, is an emerging multidisciplinary field involving biology, medicine, and engineering that is likely to revolutionize the way we improve the health and quality of life for millions of people worldwide by restoring, maintaining, or enhancing tissue and organ function[49]. Today, the field of tissue engineering has established the essential foundations for the design and fabrication of neotissues in two or three dimensions for clinical use[50].

The main requirements for producing an engineered tissue are the appropriate levels and sequencing of regulatory signals, the presence of responsive progenitor cells, an appropriate extracellular matrix or carrier construct, and an adequate blood supply. Recent advances in our knowledge concerning growth factors and biodegradable polymer constructs (scaffolds) have made possible the successful tissue engineering of cartilage, bone, and related tissues[51].

## SCAFFOLDS FOR DENTAL TISSUE ENGINEERING

The extracellular matrix (ECM) is a biologically active tissue that affects the cellular physiology of an organism, as well as some other processes, such as cell adhesion, migration, proliferation, and differentiation[52]. The main function of the scaffolds and carriers is to imitate the ECM[52]. Both biologically derived and synthetic biomaterials play an important role in the treatment of disease and the improvement of health care[53].

Polymers are often selected for the fabrication of scaffolds for the regeneration of dental tissue because their biological, chemical, and mechanical properties can be controlled. Polymers can be classified as natural or synthetic materials. Natural polymers (such as collagen, chitosan, silk, and fibrin) and synthetic polymers (such as polyglycolic acid [PGA], poly[lactic-co-glycolic acid] [PLGA], and polyglycerol sebacate [PGS]) are commonly used as scaffolds for tissue engineering[54,55].

This biomimetic strategy offers the possibility for incorporating and delivering drugs or signaling molecules via the implant surfaces. The optimization of variables, such as pore size, orientation, porosity, and interconnectivity, are still technically challenging issues. The effects of these variables, as well as the composition and structure of scaffold materials, on cell behavior and tissue formation are extensively studied[56].

Bioactive molecules incorporated into a bioresorbable scaffold are generally released by a diffusion-controlled mechanism that is regulated and controlled by the pore sizes. The rate of growth factor release also depends on the type and rate of degradation of the scaffold, and the rate of growth factor diffusion through pores of the scaffolds. Development of suitable controlled release of the bioactive molecule devices and/or alternative mode of delivery is needed[57].

Several requirements of the scaffolds are necessary for the growth of the tissues. As the cells grow and form their own structure, the scaffold should degrade so that it does not interfere with tissue growth. Thus, the degradation rate should be similar to the growth rate of the cells. Furthermore, during the degradation, it cannot release any toxins nor have any significant negative effects on cell growth or on the human body. The scaffold should have a large surface-area-to-volume ratio and should be highly porous so that nutrients and waste products can be circulated to and from the cells. A failure of the material under load may cause improper tissue growth or tearing of the scaffold; thus, the bioscaffold should have sufficient mechanical strength to withstand the stresses[58].

Tooth-like tissues have been generated by the seeding of different cell types on biodegradable scaffolds. A common methodology is to harvest cells, expand and differentiate cells *in vitro*, seed cells onto scaffolds, and implant them *in vivo*; in some cases, the scaffolds are reimplanted into an extracted tooth socket or the jaw[56].

## ENAMEL REGENERATION

Enamel is the hardest mineralized tissue in the human body. It is comprised of nanorod-like hydroxyapatite crystals arranged into a highly organized microarchitectural unit called an enamel prism[59]. The ameloblasts, together with amelogenin and ameloblastin proteins, are involved in the process of production of the hardest human tissue and the optimal protective shell of the tooth. Ameloblasts are lost with tooth eruption, thus making enamel a nonregenerating tissue[60,61].

Previous attempts to engineer enamel focused on chemical synthesis. Chen and collaborators synthesized and modified the hydroxyapatite nanorod surface with monolayers of surfactants to create specific surface characteristics that allowed the nanorods to self-assemble into an enamel prism-like structure at a water/air interface. The size of the synthetic hydroxyapatite nanorods can be controlled, and synthesized nanorods were similar in size to both human and rat enamel crystals[62]. DPSC showed biocompatibility when cultured on the produced crystals[63]. The direct growth of human enamel-like structures on the human tooth was obtained by using fluorapatite/phosphoric acid pastes. The newly formed calcium phosphate crystals showed the ability to self-assemble into an ordered microstructure similar to those seen in human enamel[59].

The use of dental and nondental stem cells also gave some positive results in the attempt to regenerate enamel tissue. Recently, BMSC were shown to acquire ameloblast characteristics when interacting with the dental mesenchyme[64].

Dental pulp cells cocultured with human fetal oral epithelial cells showed expression of amelogenin after 3 days, while oral epithelial cells alone were amelogenin immunonegative. These results imply the potential use of this heterogeneous population of cells in cell-mediated enamel tissue engineering[65].

It was reported that the culturing of murine embryonic stem cells (mESC) in ameloblast serum-free conditioned medium induces their differentiation toward dental epithelial cell lineages. After culturing these cells for 14 days in the differentiation-inducing media, the expression of ameloblast-specific proteins, such as cytokeratin 14, ameloblastin, and amelogenin, was markedly higher in mESC obtained with embryoid body (EB) formation than in mESC obtained without EB formation[66].

## DENTAL-PULP COMPLEX REGENERATION

The dental pulp complex contains mesenchymal stem/progenitor cells that have the potential to regenerate and/or repair the dentin-pulp complex after mild injury. After mild injury, pulp may respond by the activation of heat-shock proteins, while in the case of severe injury, odontoblasts and pulp cells under lesion may die. However, under appropriate conditions, a pool of stem cells inside the pulp can be activated and differentiate into odontoblasts[67,68]. These new cells will secrete a tertiary dentin matrix, which is also known as reparative dentin. If the injury is mild, primary odontoblasts and pulp cells may survive and respond by forming a deposition of a reactionary dentin matrix, a variant of tertiary dentin[69,70].

For years, calcium hydroxide was used as the gold standard for direct pulp capping. Currently, there are three different approaches for triggering pulp healing. The first is to use mineral calcium phosphates to enhance the formation of a reparative dentinal bridge, the second is to use bioactive ECM molecules that may be implicated directly in pulp mineralization or act indirectly on the commitment and differentiation of pulp progenitors, and third is to use stem cells identified within the dental pulp[71]. The importance of pulp regeneration therapy is to overcome the limitations of conventional therapy in order to induce reparative dentinogenesis.

The dentin matrix contains growth factors, especially those of the TGF-β superfamily, which can participate in tooth regeneration[72,73]. Odontoblasts secrete these growth factors, which become sequestered in the dentin matrix and remain protected there in their bioactive state until the matrix is affected by carious or other injury lesion[74,75]. When disassociated from the matrix, these molecules may diffuse to the cells of the pulp, and are available to participate in the regenerative events after tissue injury[76]. Many studies suggest that both *in vitro* and *in vivo* growth factors can induce regenerative events in the dentin-pulp complex in a similar way to dentin matrix extracts naturally containing these growth factors[74,77,78,79]. Dentin regeneration on amputated pulp was reported to be regulated by adjusting the release dosage of FGF-2 incorporated in biodegradable gelatin hydrogels[80].

According to some studies, gene therapy with bone morphogenetic proteins (BMP) can be used for dentin regeneration. BMP are involved in tooth development, and the expression of BMP is increased during terminal differentiation of odontoblasts[81]. Studies show that BMP-2 induce the mRNA expression of sialophosphoprotein, the differention marker of odontoblasts, and also induce a large amount of reparative dentin on the amputated pulp *in vivo*[82]. It has been suggested that BMP-2 may regulate the differentiation of pulp cells into odontoblastic lineage and stimulate reparative dentin formation. Calcitonin gene-related peptide and BMP-2 were demonstrated to promote reparative tertiary dentin or osteodentin formation in ferret canines[83].

The use of tissue engineering techniques also showed some results in the dentin-pulp complex regeneration. Stem/progenitor cells from canine pulp showed the potential utility as a source of cells for total pulp regeneration, with angiogenesis and vasculogenesis[84].

The implantation of DPSC seeded on a collagen scaffold, and combined with dentin matrix protein 1 (DMP1) in the site of injury, can induce an organized matrix formation similar to that of pulpal tissue, which might lead to hard tissue formation[85].

DPSC, isolated from rabbit teeth and seeded onto a PLGA polymer scaffold, which were subsequently transplanted subcutaneously in the rabbits, showed the ability to form osteodentin-like and tubular-like dentin structures[86].

Dentin-bridge formation in teeth was also reported following the transplantation of DPSC seeded on hydroxyapatite/tricalcium phosphate (HA/TCP) scaffolds[87].

It was reported that treated dentin matrix (TDM) can serve as both scaffold and inductive microenvironment, and DFPC is a suitable cell type for complete and prefabricated shaped dentin regeneration. TDM expressed type I collagen, DMP1, bone sialoprotein (BSP), dentin sialophosphoprotein (DSP), osteopontin (OPN), and transforming growth factor (TGF)-b1, decorin, and biglycan. Under the effect of these factors, DFPC showed the ability to differentiate into functional odontoblasts, which is key for dentin regeneration. DFPC, induced by TDM, expressed osteocalcin, BSP, type I collagen, OPN, osteonectin, and alkaline phosphatase, which are all expressed by odontoblasts. Most importantly, this study showed that induced DFPC strongly expressed type I collagen and secreted a rich matrix, which is also one of the typical features of odontoblasts. During dentin regeneration, phosphoproteins, DSP, decorin, biglycan, and TGF-b1 interact with each other to form predentin, which subsequently mineralizes to form dentin[88].

## REGENERATION OF PERIODONTAL TISSUE

The periodontium is composed of cementum, PDL, alveolar bone, and gingiva. Periodontium surrounds, supports, protects, and anchors the tooth[89]. Periodontitis is an inflammatory process, caused by the periodontal microbial biofilm (dental plaque), which progressively destroys the supporting structures of the teeth or periodontal attachment, and leads to tooth loss[89].

Predicable regeneration of the periodontal apparatus following periodontal disease has long been the main goal of periodontal therapy[90]. Periodontal tissue regeneration requires the restoration of alveolar bone height in relation to the cemento-enamel junction, regeneration of gingival connective tissue destroyed by inflammation, formation of new acellular and cellular cementum on previously exposed root surfaces, and synthesis of a functional PDL[91]. Numerous clinical techniques, including bone grafts, root surface conditioning, guided tissue regeneration, barrier membranes, and various growth factors, have been utilized in an attempt to achieve periodontal regeneration[92]. However, these therapeutic measures proved unable to achieve controlled and predictable regeneration[93].

The recent discovery of stem cells in periodontal tissue and the outstanding progress in biomaterial research opened the possibility for new approaches in the treatment of periodontal disease. Tissue engineering strategies for periodontal regeneration using stem cells residing within the periodontium, grown in appropriate scaffolds and subsequently implanted into the defect, may help to overcome many limitations with current therapeutic strategies[94]. Bearing in mind that wound healing is a complex process, comprising the complex network of interactions among cells, signaling molecules (growth factors, polynucleotides, cytokines, etc.), and ECM, it is of great importance to provide the appropriate signals that would recruit and stimulate the cells seeded on scaffolds[91]. These factors need to be precisely coordinated both temporally and spatially. The unpredictability of current available regeneration techniques is attributed to the lack of an optimal microenvironment[93].

Growth factors are natural cell products that are released or activated when cell division is needed[95]. They also have a role in the regulation of cell activity, chemotaxis, and/or cell differentiation[96]. The growth factors that were reported to have an important role in periodontal tissue reparation and regeneration include PDGF (platelet-derived growth factor-BB), IGF-I (insulin-like growth factor-1), basic FGF-2, TGF-1, BMP-2, BMP-4, BMP-7 and BMP-12, and enamel matrix derivative (EMD). In addition, PDGF, BMP-2, and BMP-7 have been shown to promote peri-implant bone regeneration[56]. Bone morphogenetic proteins are believed to be responsible for the initiation of cementogenesis and regulation of the assembly of a functionally oriented PDL system[97]. BMP showed the greatest potential in critical-size defects, as they are potent osteoinductive mediators. The combination of PDGF and IGF-I demonstrated the stimulation of alveolar bone repair in patients with severe periodontal disease[98]. PDGF has also been used in association with allogenic bone grafts, and was able to induce substantial attachment gain and probing depth reduction during the treatment of Class II furcation and intrabony defects[99]. Topical application of FGF-2 to the experimentally prepared periodontal tissue defects in beagle dogs and nonhuman primates induced significant periodontal tissue regeneration with new cementum and new alveolar bone formation[100]. Even more, there is evidence that suggests that the combination of β-TCP and FGF-2 can enhance bone and cementum formation[101].

It was shown that cementoblasts exhibit very high sensitivity for the concentration of inorganic phosphate in ECM. The animals with low extracellular pyrophosphate concentration have the increased cementum thickness, while high extracellular pyrophosphate concentration leads to the development of defective cementum and the absence of acellular cementum[102,103]. It is believed that pyrophosphate regulates the cementoblast secretion after the completion of cementum formation by decreasing the levels of transcripts for mineralization genes, such as BSP and osteocalcin[104].

Enamel matrix proteins (EMP), produced by Hertwig's epithelial sheath, play an important role in cementogenesis and in the development of the periodontal attachment apparatus. There is some evidence that these proteins also play a role in regeneration of periodontal tissues after periodontal therapy. *In vitro* studies have shown that EMP added to cultures of periodontal fibroblasts results in enhanced proliferation, protein and collagen production, and promotes mineralization. It is believed that EMP act as a matrix enhancement factor, creating a positive environment for cell proliferation, differentiation, and matrix synthesis[93].

P-15 is a peptide containing 15 amino acids that mimics the cell binding part of the α1 chain of type I collagen. P-15 has been shown to have the ability to enhance the rate of attachment and migration of periodontal cells on root surfaces. Clinical results indicate that the commercially available combination of P-15 and a bovine-derived hydroxyapatite matrix shows significant improvement of clinical and radiographic parameters when compared with other methods in the treatment of intrabony defects[105].

PRP is a platelet concentrate that contains a number of different growth factors, including PDGF, TGF-b, and IGF, which have been shown to have a positive effect on periodontal wound healing[106]. As autologous material is being used for PRP, the risks for the patients are minimal. The use of PRP in combination with grafts for the treatment of intrabony defects resulted in contradictory results ranging from a significant enhancement attachment gain to no effect. These discrepancies could be explained by differences in the methods used to prepare PRP, which might affect the content of platelets and inflammatory cytokines, as well as lead to contamination of PRP with leukocytes and erythrocytes[105].

The possibility of a stem cell–based tissue engineering approach to achieve periodontal regeneration is supported by animal studies demonstrating that PDL cells cultured *in vitro* can be successfully reimplanted into periodontal defects in order to promote periodontal regeneration[107]. The use of purified stem cells for tissue engineering approaches to facilitate periodontal regeneration has been widely investigated. Transplantation of autologous BMSC in combination with atelocollagen into class III defects in dogs has been shown to regenerate cementum, PDL, and alveolar bone[108]. *Ex vivo* expanded PDLSC cotransplanted with HA/TCP particles into nude rats are capable of forming cementum/PDL-like structures[109]. Cells isolated from the root apical papilla of human teeth and PDLSC can be combined to regenerate the root/periodontal structure[50]. Apical tooth germ cell–conditioned medium, which contains multiple signals and molecules, is able to create a highly cementogenic microenvironment and induce differentiation of PDLSC along the cementoblastic lineage. This was confirmed by *in vivo* transplantation of cocultured PDLSC. It was shown that PDLSC possess the tissue-regenerative capacity to produce cementum/PDL-like structures, characterized by a layer of cementum-like mineralized tissues and associated PDL-like collagen fibers connecting with the newly formed cementum-like deposits[108].

A lot of attention has been dedicated to suitable scaffolds for periodontal tissue engineering. The scaffolds used so far in periodontal tissue regeneration include allografts, xenografts, alloplasts, polymers, and collagens[110]. All these scaffolds are osteoconductive and exhibit two important limitations for use in periodontal tissue engineering: they are different from natural periodontal tissue, which is composed of soft and hard tissue, and the techniques used for their fabrication do not allow the precise control of their technical characteristics (pore size, special distribution of pores, pores geometry)[111]. Recently, new techniques, such as rapid prototyping for the fabrication of complex 3D polymer scaffolds, were introduced. The main advantage of 3D scaffolds is that it is possible to control the matrix architecture precisely[111]. Another technique used to improve the scaffold properties involves polymer surface modification performed by treatments with alkali and the attachment of peptides[110].

Single recombinant amelogenin protein (rHAM(+)) was shown to have the ability to induce *in vivo* regeneration of all tooth-supporting tissues after creation of experimental periodontitis in a dog model. Amelogenin expression was detected in normal and regenerating cells of the alveolar bone, PDL, cementum, and in bone marrow stromal cells. Amelogenin expression was highest in areas of high bone turnover and activity. rHAM(+) induced, directly or indirectly, significant recruitment of mesenchymal progenitor cells, which later differentiated to form the regenerated periodontal tissues[112]. The leucine-rich amelogenin peptide (LRAP), a split variant of full-length amelogenin, was also shown to be capable of inducing bone formation. LRAP also has the ability to control osteoclastogenesis through the RANKL pathway, thus protecting the cementum[113].

Another possible option for PDL regeneration is gene therapy that comprises the insertion of genes into an individual's cells in order to promote a specific biological effect. Targeting cells for gene therapy requires the use of vectors or direct delivery methods to transfect them[56]. In periodontal regeneration, gene therapy seeks to optimize the delivery of agents, such as growth factors, to periodontal defects so that the limitations associated with topical application can be avoided[114]. Two major strategies for delivering therapeutic transgenes into human recipients are (1) direct infusion of the gene of interest using viral or nonviral vectors *in vivo* and (2) introduction of the gene into delivery cells (often a stem cell) *ex vivo* followed by transfer of the delivery cells back into the body[115]. The treatment of periodontal defects with adenovirus (Ad)–encoding PDGF-B exhibited strong regeneration of bone and cementum. Compared with the group of control vectors, it showed fourfold increases in bridging bone and sixfold increases in tooth-lining cemental repair[116]. Recent findings in rats have revealed sustained transgene expression for up to 10 days at Ad-BMP-7 treated sites, and enhanced bone and cementum regeneration at Ad-BMP-7 and Ad-PDGF-treated sites beyond that of control vectors[116,117]. Further research is needed to identify potential risks of viral recombination and immune responses towards viral antigens that could potentially restrain the progress of gene therapy in treating periodontal diseases[118]. Recently, a nonviral gene delivery approach with nano/microbubbles and ultrasound showed promising results in therapy for periodontal disease involving alveolar bone resorption[119] .

## TOOTH REGENERATION

The replacement of lost teeth by whole tooth regeneration is one of the main goals of dental technology[120]. The progress made in the tissue engineering field has offered the possibility for biological replacement therapy of affected tissues and organs. Our current knowledge on mechanisms underlying tooth development and on the biomaterials has opened the door for the whole new approach in regenerative dentistry. Biological tooth regeneration is still, however, not possible in everyday clinical work, but the results are promising.

There are currently two approaches in tooth regeneration. One is based on the use of scaffolds seeded with stem cells (scaffold-based tooth regeneration), while the other is based on the use of stem cells without scaffolds (scaffold-free tooth regeneration).

Significant progress towards successful tooth regeneration has been achieved by Young et al., using porcine tooth bud cells on PLGA scaffolds that were implanted in immunodeficient rats[121]. Although their technique produced all tooth structures except periodontium, regeneration of a complete tooth was not achieved. However, this demonstrated that the regeneration of the tooth may be possible[121]. Their subsequent research, using porcine tooth bud cells seeded on PDGA and transplanted into the omentum of athymic adult rats, resulted in the creation of the tissue that contained dentin, cementum, pulp, and the PDL[122]. Xu and coworkers seeded rat tooth bud cells on silk fibroin scaffolds with two different pore sizes that were either used as fabricated or treated with the RGD (arginine-glycine-aspartic acid peptide) binding peptide. These tissue-engineered constructs were placed in the omenta of athymic adult rats. The larger pore sizes, as well as scaffolds treated with RGD, resulted in more mineralized osteodentin-like tissue[123]. Postnatal rat tooth bud cells seeded onto PGA/PLLA (polyglycolate/poly-L-lactate) and PLGA scaffolds and implanted and grown in the mandible can form organized bioengineered dental tissues containing dentin, enamel, pulp, and PDL. Bioengineered mandibular implant tooth tissues expressed characteristic enamel and dentin proteins, and exhibited morphological and histological appearances typical of naturally formed dentin and enamel[124].

Transplantation of a root-shaped HA/TCP block loaded with swine-SCAP, which was coated with Gelform containing PDLSC, into the extraction socket from a minipig lower incisor showed the successful regeneration of the root/periodontal structure over which a porcelain crown could have been placed[125]. Tooth buds in mice (E14.5) that were dissociated into single cells, seeded onto a collagen-coated PGA fiber mesh, and transplanted into the kidney capsule of male adult mice showed the normal enamel prism-like organization in the newly produced enamel after 14 days. All biological teeth produced by this method, in which E14 mice molar tooth buds are used, exhibited normal crown structures composed of enamel and dentin, covering pulp-like tissues. This study showed that completely dispersed tooth bud cells have the capacity to produce teeth even though scaffolds were used. High-density seeding of isolated porcine mesenchymal cells onto the scaffold, followed by the placing of isolated porcine dental epithelial cells facilitated formation of tooth-like structures, both *in vivo* and *in vitro*, that were more uniform in shape than achieved previously[121].

However, the scaffold approach fails to produce the appropriate size of the tooth. This size limitation may be a consequence of the animal model or directly related to mass transfer[78]. Animal studies using scaffold-based approaches often rely on *in vivo* maturation of a small scaffold in the renal capsule or omentum, followed by implantation into the jaw to support and develop a tooth-like structure[122,126]. *In vitro* approaches generally rely on perfusion or flow-based bioreactors that facilitate a deeper exchange of molecules within the scaffold[127,128].

Scaffold-free tooth engineering comprises cell-tissue recombination. This involves the combination of intact embryonic epithelial tissue with isolated dental mesenchymal cells or other mesenchymal stem cells[126,129]. Recently, E14 tooth germs have been dissociated and reassociated with intact dental mesenchyme in combination with isolated dental epithelial cell populations, resulting in tooth histogenesis *in vitro*[130]. These regenerated teeth had normally shaped crowns, compared to those produced in studies using postnatal tooth cells[131,132]. This suggests that postnatal tooth cells have already lost essential factors involved in regulation of tooth shape. However, these approaches are difficult to apply clinically for adults, in whom teeth have already completely formed, so the need remains to improve the regulation of tooth shape in teeth regenerated from postnatal cells[120].

The further investigations using BMP-4 showed that the dissected tooth germ from the mouse transplanted in the diastema and cultured with administration of BMP-4 could lead to the mature development of the dental structures. The bioengineered tooth displayed increased maturation of cusp and enamel matrix[133].

Recently, it was reported that the effects of mechanical stress on regenerating tooth cells before transplantation increases the duration of tooth regeneration[134].

Reassociations performed using (1) dissociated epithelial cells and dissociated mesenchymal cells, (2) epithelial cells and an intact dental mesenchyme, and (3) one intact dental epithelium with different numbers of dental mesenchymal cells, which were cultured *in vitro* for 6–8 days before they were implanted under the skin behind the ears of adult mice, showed the development of the crown, root, and periodontal fibers in contact with newly formed bone. This clinically feasible approach thus proposes a two-step implantation-transplantation strategy. An initial temporary ectopic implantation under the skin of the host to reduce immunoreaction also allows further development of the crown as well as the formation of root tissues, the periodontium, and surrounding bone[135].

Recently, there was an attempt to bioengineer teeth using a spontaneously immortalized mesenchymal cell line. Odontoblast-lineage cells (OLC) were reassociated with competent dental epithelium isolated from E14.5 mice. A novel 3D organ germ culture method was applied to nurture the constructs *in vitro*. Additionally, recombinants were transplanted under the kidney capsule in host animals for 2 weeks. Transplants developed into tooth tissues in one-third of the cases[136].

## CHALLENGES AND FUTURE DIRECTION

Despite the progress in stem cell biology and tissue engineering techniques, we are still far from application of dental stem cells in clinical practice. There are still a number of general problems associated with stem cell therapies, listed below, as suggested by Bhatt and Le[137]:

1. The problem is how to obtain the sufficient number of stem cells for the treatment without causing major morbidities to the harvesting site.
2. The transplanted cells tend to migrate or die, resulting in a small percentage of donor cells actually engrafted at the recipient site.
3. The lineage-specific stem cells, which have undergone multiple cell divisions for *ex vivo* expansion, may not be capable to maintain long-term viability or differentiation capacities necessary for the success of tissue regeneration.
4. There is concern about the short- and long-term effects of graft vs. host disease.
5. Our knowledge about potential chromosomal instability and tumorigenicity of dental stem cells is not sufficient.

Beside the above-mentioned limitations, there are also specific issues related to dental tissue engineering. Nakahara and Ide point out the most important of them[125]:

1. Current tissue engineering techniques cannot mimic the process of correct tooth morphogenesis.
2. There is no suitable substitute for the embryonic oral epithelium, which has a unique set of signals for odontogenesis.
3. There is no embryonic environment that enables bone marrow cells to differentiate into tooth germ cells.
4. There are problems of immune rejection and ethical issues on the use of human embryos.

Even with these problems solved, there is still the question of the ideal scaffolds and the induction of adequate neovascularization. Further research in this area is needed in order to surpass all these obstacles, as well as cooperation with other disciplines. The introduction of dental stem cell–based therapy in clinical work would revolutionize regenerative dentistry, bringing a whole new concept for the treatment of tooth loss, caries, and periodontitis, as well as in different conditions of the craniofacial region.

## ACKNOWLEDGMENTS

This work was supported by a grant, No 145004, from the Ministry of Science and Technological Development of Serbia. The authors declare no conflict of interests.

## References

1. Honda. M.J., Fong, H., Iwatsuki, S., Sumita, Y., and Sarikaya M. (2008) Tooth-forming potential in embryonic and postnatal tooth bud cells. *Med. Mol. Morphol.* **41**, 183–192.
2. Yen, A.H.H. and Sharpe, P.T. (2008) Stem cells and tooth tissue engineering. *Cell Tissue Res.* **331**, 359–372.
3. Thesleff, I. (2003) Epithelial-mesenchymal signalling regulating tooth morphogenesis. *J. Cell Sci.* **116**, 1647–1648.
4. Tissier-Seta, J.P., Mucchielli, M.L., Mark, M., Mattei, M.G., Goridis, C., and Brunet, J.F. (1995) Barx1, a new mouse homeodomain transcription factor expressed in cranio-facial ectomesenchyme and the stomach. *Mech. Dev.* **51,** 3–15.
5. Mitsiadis, T.A., Angeli, I., James, C., Lendahl, U., and Sharpe, P.T. (2003) Role of Islet1 in the patterning of murine dentition. *Development* **130,** 4451–4460.
6. Peters, H. and Balling, R. (2000) Teeth: where and how to make them. *Trends Genet.* **15,** 59–65.
7. St Amand, T.R., Zhang, Y.D., Semina, E.V., Zhao, X., Hu, Y., Nguyen, L., Murray, J.C., and Chen, Y. (2000) Antagonistic signals between BMP4 and FGF8 define the expression of Pitx1 and Pitx2 in mouse tooth forming anlage. *Dev. Biol.* **217,** 323–332.
8. Vainio, S., Karavanova, I., Jowett, A., and Thesleff, I. (1993) Identification of BMP-4 as a signal mediating secondary induction between epithelial and mesenchymal tissues during early tooth development. *Cell* **75,** 45–58.
9. Thesleff, I. and Mikkola, M. (2002) The role of growth factors in tooth development. *Int. Rev. Cytol.* **217,** 93–135.
10. Zhang, Y.D., Zhang, Z.Y., Zhao, X.A., Yu, X., Hu, Y., Geronimo, B., Fromm, S.H., and Chen, Y.P. (2000) New function of BMP4: dual role for BMP4 in regulation of Sonic hedgehog expression in the mouse tooth germ. *Development* **127,** 1431–1443.
11. Vaahtokari, A., Aberg, T., Jernvall, J., Keränen, S., and Thesleff, I. (1996) The enamel knot as a signaling center in the developing mouse tooth. *Mech. Dev.* **54**, 39–43.
12. Thesleff, I. and Pispa, J. (1998) The teeth as models for studies on the molecular basis of the development and evolution of organs. In *Molecular Basis of Epithelial Appendage Morphogenesis*. Chuong, C.M., Ed. Landes Bioscience, Austin, TX. Pp. 157–179.
13. Thesleff, I., Keranen, S., and Jernvall, J. (2001) Enamel knots as signaling centers linking tooth morphogenesis and odontoblast differentiation. *Adv. Dent. Res.* **15**, 14–18.
14. Slavkin, H.C., Bessem, C., Fincham, A.G., Bringas, P.J., Santos, V., Snead, M.L., and Zeichner-David, M. (1989) Human and mouse cementum proteins immunologically related to enamel proteins. *Biochim. Biophys. Acta* **991**, 12–18.
15. Zeichner-David, M., Oishi, K., Su, Z., Zakartchenko, V., Chen, L.S., Arzate, H., and Bringas, P.J. (2003) Role of Hertwig's epithelial root sheath cells in tooth root development. *Dev. Dyn.* **228**, 651–663.
16. Lézot, F., Davideau, J.L., Thomas, B., Sharpe, P., Forest, N., and Berdal, A. (2000) Epithelial Dlx-2 homeogene expression and cementogenesis. *J. Histochem. Cytochem.* **48**, 277–284.
17. Steele-Perkins, G., Butz, K.G., Lyons, G.E., Zeichner-David, M., Kim, H.J., Cho, M.I., and Gronostajski, R.M. (2003) Essential role for NFI-C/CTF transcription-replication factor in tooth root development. *Mol. Cell. Biol.* **23**, 1075–1084.
18. Cho, M.I. and Garant, P.R. (1996) Expression and role of epidermal growth factor receptors during differentiation of cementoblasts, osteoblasts, and periodontal ligament fibroblasts in the rat. *Anat. Rec.* **245**, 342–360.
19. McCulloch, C.A. (2000) Proteomics for the periodontium: current strategies and future promise. *Periodontol. 2000* **40**, 173–183.
20. Yamada, S., Tomoeda, M., Ozawa, Y., Yoneda, S., Terashima, Y., Ikezawa, K., Ikegawa, S., Saito, M., Toyosawa, S., and Murakami, S. (2007) PLAP-1/asporin, a novel negative regulator of periodontal ligament mineralization. *J. Biol. Chem.* **282**, 23070–23080.

21. Kruzynska-Frejtag, A., Wang, J., Maeda, M., Rogers, R., Krug, E., Hoffman, S., Markwald, R.R., and Conway, S.J. (2004) Periostin is expressed within the developing teeth at the sites of epithelial–mesenchymal interaction. *Dev. Dyn.* **229**, 857–868.
22. Kii, I., Amizuka, N., Minqi, L., Kitajima, S., Saga, Y., and Kudo, A. (2006) Periostin is an extracellular matrix protein required for eruption of incisors in mice. *Biochem. Biophys. Res. Commun.* **342**, 766–772.
23. Kitagawa, M., Kudo, Y., Iizuka, S., Ogawa, I., Abiko, Y., Miyauchi, M., and Takata, T. (2006) Effect of F-spondin on cementoblastic differentiation of human periodontal ligament cells. *Biochem. Biophys. Res. Commun.* **349**, 1050–1056.
24. Parker, G.C., Anastassova-Kristevam, M., Broxmeyer, H.E., Dodge, W.H., Eisenberg, L.M., Gehling, U.M., Guenin, L.M., Huss, R., Moldovan, N.I., Rao, M., Srour, E.F., and Yoder, M.C. (2004) Stem cells: shibboleths of development. *Stem Cells Dev.* **13**, 579–584.
25. Caplan, A.I. (1991) Mesenchymal stem cells. *J. Orthop. Res.* **9**, 641–650.
26. Morsczeck, C., Schmalz, G., Reichert, T.E., Völlner, F., Galler, K., and Driemel, O. (2008) Somatic stem cells for regenerative dentistry. *Clin. Oral. Investig.* **12**, 113–118.
27. Friedenstein, A.J., Chailakhjan, R.K., and Lalykina, K.S. (1970) The development of fibroblast colonies in monolayer cultures of guinea-pig bone marrow and spleen cells. *Cell Tissue Kinet.* **3**, 393–403.
28. Pittenger, M.F., Mackay, A.M., Beck, S.C., Jaiswal, R.K., Douglas, R., Mosca, J.D., Moorman, M.A., Simonetti, D.W., Craig, S., and Marshak, D.R. (1999) Multilineage potential of adult human mesenchymal stem cells. *Science* **284**, 143–147.
29. Prockop, D.J. (1997) Marrow stromal cells as stem cells for nonhematopoietic tissues. *Science* **276**, 71–74.
30. Mao, J.J., Giannobile, W.V., Helms, J.A., Hollister, S.J., Krebsbach, P.H., Longaker, M.T., and Shi, S. (2006) Craniofacial tissue engineering by stem cells. *J. Dent. Res.* **85**, 966–979.
31. Maria, O.M., Khosravi, R., Mezey, E., and Tran, S.D. (2007) Cells from bone marrow that evolve into oral tissues and their clinical applications. *Oral Dis.* **13**, 11–16.
32. Murray, P.E., Garcia-Godoy, F., and Hargreaves, K.M. (2007) Regenerative endodontics: a review of current status and a call for action. *J. Endod*. **33**, 377–390.
33. Ohazama, A., Modino, S.A., Miletich, I., and Sharpe, P.T. (2004) Stem-cell-based tissue engineering of murine teeth. *J. Dent. Res.* **83**, 518–522.
34. Sloan, A.J. and Smith, A.J. (2007) Stem cells and the dental pulp: potential roles in dentine regeneration and repair. *Oral Dis.* **13**, 151–157.
35. Song, A.M., Shu, R., Xie, Y.F., Song, Z.C., Li, H.Y., Liu, X.F., and Zhang, X.L. (2007) A study of enamel matrix proteins on differentiation of porcine bone marrow stromal cells into cementoblasts. *Cell Prolif.* **40**, 381–396.
36. Hu, B., Unda, F., Bopp-Kuchler, S., Jimenez, L., Wang, X.J., Haïkel, Y., Wang, S.L., and Lesot, H. (2006) Bone marrow cells can give rise to ameloblast-like cells. *J. Dent. Res.* **85**, 416–421.
37. Gronthos, S., Mankani, M., Brahim, J., Robey, P.G., and Shi, S. (2000) Postnatal human dental pulp stem cells (DPSC) in vitro and in vivo. *Proc. Natl. Acad. Sci. U. S. A.* **97**, 13625–13630.
38. Miura, M., Gronthos, S., Zhao, M., Lu, B., Fisher, L.W., Robey, P.G., and Shi, S. (2003) SHED: stem cells from human exfoliated deciduous teeth. *Proc. Natl. Acad. Sci. U. S. A.* **100**, 5807–5812.
39. Shi, S. (2001) Comparison of human dental pulp and bone marrow stromal stem cells by cDNA microarray analysis. *Bone* **29,** 532–539.
40. Gronthos, S., Brahim, J., Li, W., Fisher, L.W., Cherman, N., Boyde, A., DenBesten, P., Gehron Robey, P., and Shi, S. (2002) Stem cell properties of human dental pulp stem cells. *J. Dent. Res.* **81**, 531–535.
41. Papaccio, G., Graziano, A., d'Aquino, R., Graziano, M.F., Pirozzi, G., Menditti, D., De Rosa, A., Carinci, F., and Laino, G. (2006) Long-term cryopreservation of dental pulp stem cells (SBP-DPSC) and their differentiated osteoblasts: a cell source for tissue repair. *J. Cell. Physiol.* **208**, 319–325.
42. Shi, S. and Gronthos, S. (2003) Perivascular niche of postnatal mesenchymal stem cells in human bone marrow and dental pulp. *J. Bone Miner. Res.* **18**, 696–704.
43. Gay, I., Chen, S., and Macdougall, M. (2007) Isolation and characterization of multipotent human periodontal ligament stem cells. *Orthod. Craniofac. Res.* **10**, 149–160.
44. Seo, B.M., Miura, M., Gronthos, S., Bartold, P.M., Batouli, S., Brahim, J., Young, M., Roby, P.G., Wang, C.Y., and Shi, S. (2004) Investigation of multipotent postnatal stem cells from human periodontal ligament. *Lancet* **364**, 149–155.
45. Morsczeck, C., Moehl, C., Gotz, W., Heredia, A., Schäffer, T.E., Eckstein, N., Sippel, C., and Hoffmann, K.H. (2005) In vitro differentiation of human dental follicle cells with dexamethasone and insulin. *Cell Biol. Int*. **29**, 567–575.
46. Morsczeck, C., Gotz, W., Schierholz, J., Zeilhofer, F., Kühn, U., Möhl, C., Sippel, C., and Hoffmann, K.H. (2005) Isolation of precursor cells (PCs) from human dental follicle of wisdom teeth. *Matrix Biol.* **24**, 155–165.
47. Morsczeck, C. (2006) Gene expression of runx2, osterix, c-fos, DLX-3, DLX-5 & MSX-2 in dental follicle cells during osteogenic differentiation in vitro. *Calcif. Tissue Int.* **78**, 98–102.
48. Sonoyama, W., Liu, Y., Fang, D., Yamaza, T., Seo, B.M., Zhang, C., Liu, H., Gronthos, S., Wang, C.Y., Shi, S., and Wang, S. (2006) Mesenchymal stem cell-mediated functional tooth regeneration in swine. *PLoS One* **1**, e79.
49. Bartold, P.M., Xiao, Y., Lyngstaadas, S.P., Paine, M.L., and Snead, M.L. (2006) Principles and applications of cell delivery systems for periodontal regeneration. *Periodontol. 2000* **41**, 123–135.

50. Lavik, E. and Langer, R. (2004) Tissue engineering: current state and perspectives. *Appl. Microbiol. Biotechnol.* **65**, 1–8.
51. Slavkin, H.C. and Bartold, P.M. (2006) Challenges and potential in tissue engineering. *Periodontol. 2000* **41**, 9–15.
52. Kleinman, H.K., Philp, D., and Hoffman, M.P. (2003) Role of the extracellular matrix in morphogenesis. *Curr. Opin. Biotechnol*. **14**, 526–532.
53. Du, C. and Moradian-Oldak, J. (2006) Tooth regeneration: challenges and opportunities for biomedical material research. *Biomed. Mater.* **1**, 10–17.
54. Vozzi, G., Flaim, C., Ahluwalia, A., and Bhatia, S. (2003) Fabrication of PLGA scaffolds using soft lithography and microsyringe deposition. *Biomaterials* **24**, 2533–2540.
55. Chevrier, A., Hoemann, C.D., Sun, J., and Buschmann, M.D. (2007) Chitosan-glycerol phosphate/blood implants increase cell recruitment, transient vascularization and subchondral bone remodeling in drilled cartilage defects. *Osteoarthritis Cartilage* **15**, 316–327.
56. Taba, M., Jin, J.Q., Sugai, J.V., and Giannobile, W.V. (2005) Current concepts in periodontal bioengineering. *Orthod. Craniofac. Res.* **8**, 292–302.
57. Leung1, L., Chan, C., Baek, S., and Naguib, H. (2008) Comparison of morphology and mechanical properties of PLGA bioscaffolds. *Biomed. Mater.* **3**, 1–9.
58. Hacking, S.A. and Khademhosseini, A. (2009) Application of microscale technologies for regenerative dentistry. *J. Dent. Res.* **88**, 409–421.
59. Wang, X., Xia, C., Zhang, Z., Deng, X., Wei, S., Zheng, G., and Chen, H. (2009) Direct growth of human enamel-like calcium phosphate microstructures on human tooth. *J. Nanosci. Nanotechnol*. **9**, 1361–1364.
60. Paine, M.L., White, S.N., Luo, W., Fong, H., Sarikaya, M., and Snead, M.L. (2001) Regulated gene expression dictates enamel structure and tooth function. *Matrix Biol.* **20**, 273–292.
61. Zaky, S.H. and Cancedda, R. (2009) Engineering craniofacial structures: facing the challenge**.** *J. Dent. Res.* **88**, 1077–1091.
62. Chen, H., Clarkson, B.H., Sun, K., and Mansfield, J.F. (2005) Self-assembly of synthetic hydroxyapatite nanorods into an enamel prism-like structure. *J. Colloid Interface Sci.* **288**, 97–103.
63. Chen, H., Tang, Z., Liu, J., Sun, K., Chang, S.R., and Peters, M.C. (2006) Acellular synthesis of a human enamel-like microstructure. *Adv. Mater.* **18**, 1846–1851.
64. Hu, B., Unda, F., Bopp-Kuchler, S., Jimenez, L., Wang, X.J., Haikel, Y., Wang, S.L., and Lesot, H. (2006) Bone marrow cells can give rise to ameloblast-like cells. *J. Dent. Res.* **85**, 416–421.
65. Coppe, C., Zhang, Y., and Den Besten, P.K. (2009) Characterisation of primary dental pulp cells in vitro. *Pediatr. Dent*. **31**, 467–471.
66. Ning, F., Guo, Y., Tang, J., Zhou, J., Zhang, H., Lu, W., Gao, Y., Wang, L., Pei, D., Duan, Y., and Jin, Y. (2010) Differentiation of mouse embryonic stem cells into dental epithelial-like cells induced by ameloblasts serum-free conditioned medium. *Biochem. Biophys. Res. Commun*. **394(2),** 342–347.
67. Kitamura, C., Kimura, K., Nakayama, T., Toyoshima, K., and Terashita, M. (2001) Primary and secondary induction of apoptosis in odontoblasts after cavity preparation of rat molars. *J. Dent .Res.* **80**, 1530–1534.
68. Kitamura, C., Ogawa, Y., Nishihara, T., Morotomi, T., and Terashita, M. (2003) Transient co-localization of c-Jun N-terminal kinase and c-Jun with heat shock protein 70 in pulp cells during apoptosis. *J. Dent. Res.* **82**, 91–95.
69. Lesot, H., Begue-Kirn, C., Kubler, M.D., Meyer, J.M., Smith, A.J., and Cassidy, N. (1993) Experimental induction of odontoblast differentiation and stimulation during reparative processes. *Cell. Mater.* **3**, 201–217.
70. Smith, A.J., Cassidy, N., Perry, H., Bègue-Kirn, C., Ruch, J.V., and Lesot, H. (1995) Reactionary dentinogenesis. *Int. J. Dev. Biol.* **39**, 273–280.
71. Goldberg, M., Six, N., Chaussain, C., DenBesten, P., Veis, A., and Poliard, A. (2009) Dentin extracellular matrix molecules implanted into exposed pulps generate reparative dentin: a novel strategy in regenerative dentistry. *J. Dent .Res.* **88**, 396–399.
72. Cassidy, N., Fahey, M., Prime, S.S., and Smith, A.J. (1997) Comparative analysis of transforming growth factor-beta (TGF-b) isoforms 1–3 in human and rabbit dentine matrices. *Arch. Oral Biol*. **42**, 219–223.
73. Finkelman, R.D., Mohan, S., Jennings, J.C., Taylor, A.K., Jepsen, S., and Baylink, D.J. (1990) Quantitation of growth factors IGF-I, SGF/IGF II and TGF-b in human dentin. *J. Bone Miner. Res.* **5**, 717–723.
74. Sloan, A.J., Perry, H., Matthews, J.B., and Smith, A.J. (2000) Transforming growth factor-b isoform expression in mature human molar teeth. *Histochem. J.* **32**, 247–252.
75. Smith, A.J., Matthews, J.B., and Hal, R.C. (1998) Transforming growth factor-b1 (TGF-b1) in dentine matrix: ligand activation and receptor expression. *Eur. J. Oral Sci.* **106**, 179–184.
76. Smith, A.J., Lumley, P.J., Tomson, P.L., and Cooper, P.R. (2008) Dental regeneration and materials—a partnership. *Clin. Oral Investig.* **12**, 103–108.
77. Hu, C.C., Zhang, C., Qian, Q., and Tatum, N.B. (1998) Reparative dentin formation in rat molars after direct pulp capping with growth factors. *J. Endodont.* **24**, 744–751.
78. Rutherford, R.B., Wahle, J., Tucker, M., Rueger, D., and Charette, M. (1993) Induction of reparative dentine formation in monkeys by recombinant human osteogenic protein-1. *Arch. Oral Biol.* **38**, 571–576.

79. Tziafas, D., Alvanou, A., Papadimitriou, S., Gasic, J., and Komnenou, A. (1998) Effects of recombinant fibroblast growth factor, insulin-like growth factor-II and transforming growth factor-β1 on dog dental pulp cells in vivo. *Arch. Oral Biol*. **43**, 431–444.
80. Ishimatsu, H., Kitamura, C., Morotomi, T., Tabata, Y., Nishihara, T., Chen, K.K., and Terashita, M. (2009) Formation of dentinal bridge on surface of regenerated dental pulp in dentin defects by controlled release of fibroblast growth factor-2 from gelatin hydrogels. *J. Endod.* **35**, 858–865.
81. Nakashima, M. and Reddi, A.H. (2003) The application of bone morphogenetic protein to dental tissue engineering. *Nat. Biotech.* **21**, 1025–1031.
82. Nakashima, M. (1994) Induction of dentin formation on canine amputated pulp by recombinant human bone morphogenetic protein (BMP)-2 and -4. *J. Dent. Res.* **73**, 1515–1522.
83. Kline, L.W. and Yu, D.C. (2009) Effects of calcitonin, calcitonin gene-related peptide, human recombinant bone morphogenetic protein-2, and parathyroid hormone-related protein on endodontically treated ferret canines. *J. Endod*. **35**, 866–869.
84. Iohara, K., Zheng, L., Ito, M., Ishizaka, R., Nakamura, H., Into, T., Matsushita, K., and Nakashima, M. (2009) Regeneration of dental pulp after pulpotomy by transplantation of CD31(-)/CD146(-) side population cells from a canine tooth. *Regen. Med.* **4**, 377–385.
85. Prescott, R.S., Alsanea, R., Fayad, M.I., Johnson, B.R., Wenckus, C.S., Hao, J., John, A.S., and George, A. (2008) In vivo generation of dental pulp-like tissue by using dental pulp stem cells, a collagen scaffold, and dentin matrix protein 1 after subcutaneous transplantation in mice. *J. Endod*. **34**, 421–426.
86. El-Backly, R.M., Massoud, A.G., El-Badry, A.M., Sherif, R.A., and Marei, M.K. (2008) Regeneration of dentine/pulp-like tissue using a dental pulp stem cell/poly(lactic-co-glycolic) acid scaffold construct in New Zealand white rabbits. *Aust. Endod. J*. **34**, 52–67.
87. Ando, Y., Honda, M.J., Ohshima, H., Tonomura, A., Ohara, T., Itaya, T., Kagami, H., and Ueda, M. (2009) The induction of dentin bridge-like structures by constructs of subcultured dental pulp-derived cells and porous HA/TCP in porcine teeth. *Nagoya J. Med. Sci.* **71**, 51–62.
88. Guo, W., He, Y., Zhang, X., Lu, W., Wang, C., Yu, H., Liu, Y., Li, Y., Zhou, Y., Zhou, J., Zhang, M., Deng, Z., and Jin, Y. (2009) The use of dentin matrix scaffold and dental follicle cells for dentin regeneration. *Biomaterials* **30(35),** 6708–6723.
89. Nanci, A. and Bosshardt, D.D. (2006) Structure of periodontal tissues in health and disease. *Periodontol. 2000* **40**, 11–28.
90 Ripamonti, U. (2007) Recapitulating development: a template for periodontal tissue engineering. *Tissue Eng.* **13**, 51–71.
91. Chen, F.M., Shelton, R.M., Jin, Y., and Chapple, I.L. (2009) Localized delivery of growth factors for periodontal tissue regeneration: role, strategies, and perspectives. *Med. Res. Rev*. **29**, 472–513.
92. Yang, Z.H., Zhang, X.J., Dang, N.N., Ma, Z.F., Xu, L., Wu, J.J., Sun, Y.J., Duan, Y.Z., Lin, Z., and Jin, Y. (2009) Apical tooth germ cell-conditioned medium enhances the differentiation of periodontal ligament stem cells into cementum/periodontal ligament-like tissues. *J. Periodont. Res.* **44**, 199–210.
93. Ivanovski, S., Gronthos, S., Shi, S., and Bartold, P.M. (2006) Stem cells in the periodontal ligament. *Oral Dis.* **12**, 358–363.
94. Bartold, P.M., McCulloch, C.A., Narayanan, A.S., and Pitaru, S. (2003) Tissue engineering: a new paradigm for periodontal regeneration based on molecular and cell biology. *Periodontol. 2000* **24**, 253–269.
95. Raja, S., Byakod, G., and Pudakalkat, P. (2009) Growth factors in periodontal Regeneration. *Int. J. Dent. Hyg.* **7**, 82–89.
96. Bosshardt, D.D. (2008) Biological mediators and periodontal regeneration: a review of enamel matrix proteins at the cellular and molecular levels. *J. Clin. Periodontol.* **35**, 87–105.
97. Ripamonti, U., Petit, J.C., and Teare, J. (2009) Cementogenesis and the induction of periodontal tissue regeneration by the osteogenic proteins of the transforming growth factor-b superfamily. *J. Periodont. Res.* **44**, 141–152.
98. Howell, T.H., Fiorellini, J.P., Paquette, D.W., Offenbacher, S., Giannobile, W.V., and Lynch, S.E. (1997) A phase I/II clinical trial to evaluate a combination of recombinant human platelet-derived growth factor-BB and recombinant human insulin-like growth factor-I in patients with periodontal disease. *J. Periodontol.* **68**, 1186–1193.
99. Camelo, M., Nevins, M.L., Schenk, R.K., Lynch, S.E., and Nevins, M. (2003) Periodontal regeneration in human Class II furcations using purified recombinant human platelet-derived growth factor-BB (rhPDGF-BB) with bone allograft. *Int. J. Periodontics Restorative Dent.* **23**, 213–225.
100. Murakami, S. (2007) Periodontal regeneration by FGF2. *Clin. Calcium* **17**, 249–255.
101. Oi, Y., Ota, M., Yamamoto, S., Shibukawa, Y., and Yamada, S. (2009) β-Tricalcium phosphate and basic fibroblast growth factor combination enhances periodontal regeneration in intrabony defects in dogs. *Dent. Mater. J.* **28**, 162–169.
102. Nociti, F.H., Jr., Berry, J.E., Foster, B.L., Gurlev, K.A., Kingsley, D.M., Takata, T., Miyauchi, M., and Somerman, M.J. (2002) Cementum: a phosphate-sensitive tissue. *J. Dent. Res.* **81**, 817–821.
103. Beertsen, W., VandenBos, T., and Everts, V. (1999) Root development in mice lacking functional tissue non-specific alkaline phosphatase gene: inhibition of acellular cementum formation. *J. Dent. Res.* **78,** 1221–1229.

104. Foster, B.L., Nociti, F.H., Jr., Swanson, E.C., Matsa-Dunn, D., Berry, J.E., Cupp, C.J., Zhang, P., and Somerman, M.J. (2006) Regulation of cementoblast gene expression by inorganic phosphate in vitro. *Calcif. Tissue Int*. **78**, 103–112.
105. Ivanovski, S. (2009) Periodontal regeneration. *Aust. Dent. J.* **54**, 118–128.
106. Okuda, K., Kawase, T., Momose, M., Murata, M., Saito, Y., Suzuki, H., Wolff, L.F., and Yoshie, H. (2003) Platelet-rich plasma contains high levels of platelet-derived growth factor and transforming growth factor-beta and modulates the proliferation of periodontally related cells in vitro. *J. Periodontol.* **74**, 849–857.
107. Hasegawa, M., Yamato, M., Kikuchi, A., Okano, T., and Ishikawa, I. (2005) Human periodontal ligament cell sheets can regenerate periodontal ligament tissue in an athymic rat model. *Tissue Eng.* **11**, 469–477.
108. Kawaguchi, H., Hirachi, A., Hasegawa, N., Iwata, T., Hamaguchi, H., Shiba, H., Takata, T., Kato, Y., and Kurihara, H. (2004) Enhancement of periodontal tissue regeneration by transplantation of bone marrow mesenchymal stem cells. *J. Periodontol.* **75**, 1281–1287.
109. Seo, B.M., Miura, M., Sonoyama, W., Coppe, C., Stanyon, R., and Shi, S. (2005) Recovery of stem cells from cryopreserved periodontal ligament. *J. Dent. Res.* **84**, 907–912.
110. Benatti, B.B., Silvério, K.G., Casati, M.Z., Sallum, E.A., and Nociti, F.H., Jr. (2007) Physiological features of periodontal regeneration and approaches for periodontal tissue engineering utilizing periodontal ligament cells. *J. Biosci. Bioeng*. **103**, 1–6.
111. Peltola, S.M., Melchels, F.P., Grijpma, D.W., and Kellomaki, M. (2008) A review of rapid prototyping technique for tissue engineering purpose. *Ann. Med*. **40**, 268–240.
112. Haze, A., Taylor, A.L., Haegewald, S., Leiser, Y., Shay, B., Rosenfeld, E., Gruenbaum-Cohen, Y., Dafni, L., Zimmermann, B., Heikinheimo, K., Gibson, C.W., Fisher, L.W., Young, M.F., Blumenfeld, A., Bernimoulin, J.P., and Deutsch, D. (2009) Regeneration of bone and periodontal ligament induced by recombinant amelogenin after periodontitis. *J. Cell. Mol. Med.* **13**, 1110–1124.
113. Hatakeyama, J., Philp, D., Hatakeyama, Y., Haruyama, N., Shum, L., Aragon, M.A., Yuan, Z., Gibson, C.W., Sreenath, T., Kleinman, H.K., and Kulkarni, A.B. (2006) Amelogenin-mediated regulation of osteoclastogenesis, and periodontal cell proliferation and migration. *J. Dent. Res.* **85,** 144–149.
114. Ramseier, C.A., Abramson, Z.R., Jin, Q., and Giannobile, W.V. (2006) Gene therapeutics for periodontal regenerative medicine. *Dent. Clin. North Am.* **50**, 245–263.
115. Zwaka, T.P. (2006) Use of genetically modified stem cells in experimental gene therapies. In Regenerative Medicine 2006. National Institutes of Health. Department of Health and Human Services, Bethesda, MD. pp. 45–52.
116. Jin, Q., Anusaksathien, O., Webb, S.A., Printz, M.A., and Giannobile, W.V. (2004) Engineering of tooth-supporting structures by delivery of PDGF gene therapy vectors. *Mol. Ther.* **9**, 519–526.
117. Dunn, C.A., Jin, Q., Taba, M., Jr., Franceschi, R.T., Bruce Rutherford, R., and Giannobile, W.V. (2005) BMP gene delivery for alveolar bone engineering at dental implant defects. *Mol. Ther.* **11**, 294–299.
118. Lin, N.H., Gronthos, S., and Bartold, P.M. (2008) Stem cells and periodontal regeneration**.** *Aust. Dent. J*. **53**,108–121.
119. Chen, R., Chiba, M., Mori, S., Fukumoto, M., and Kodama, T. (2009) Periodontal gene transfer by ultrasound and nano/microbubbles. *J. Dent. Res.* **88**, 1008–1013.
120. Honda, M.J., Tsuchiya, S., Sumita, Y., Sagara, H., and Ueda, M. (2007) The sequential seeding of epithelial and mesenchymal cells for tissue-engineered tooth regeneration. *Biomaterials* **28**, 680–689.
121. Young, C.S., Terada, S., Vacanti, J.P., Honda, M., Bartlett, J.D., and Yelick, P.C. (2002) Tissue engineering of complex tooth structures on biodegradable polymer scaffolds. *J. Dent. Res.* **81**, 695–700.
122. Young, C.S., Abukawa, H., Asrican, R., Ravens, M., Troulis, M.J., Kaban, L.B., Vacanti, J.P., and Yelick, P.C. (2005) Tissue-engineered hybrid tooth and bone. *Tissue Eng.* **11**, 1599–1610.
123. Xu, W.P., Zhang, W., Asrican, R., Kim, H.J., Kaplan, D.L., and Yelick, P.C. (2008) Accurately shaped tooth bud cell-derived mineralized tissue formation on silk scaffolds. *Tissue Eng. Part A* **14**, 549–557.
124. Duailibi, S.E., Duailibi, M.T., Zhang, W., Asrican, R., Vacanti, J.P., and Yelick, P.C. (2008) Bioengineered dental tissues grown in the rat jaw. *J. Dent. Res.* **87**, 745–750.
125. Nakahara, T. and Ide, Y. (2007) Tooth regeneration: implications for the use of bioengineered organs in first-wave organ replacement. *Hum. Cell* **20**, 63–70.
126. Ohazama, A., Modino, S.A., Miletich, I., and Sharpe, P.T. (2004) Stem-cell-based tissue engineering of murine teeth. *J. Dent. Res.* **83**, 518–522.
127. Timmins, N.E., Scherberich, A., Früh, J.A., Heberer, M., Martin, I., and Jakob, M. (2007) Three-dimensional cell culture and tissue engineering in a T-CUP (tissue culture under perfusion). *Tissue Eng.* **13**, 2021–2028.
128. Jaasma, M.J., Plunkett, N.A., and O'Brien, F.J. (2008) Design and validation of a dynamic flow perfusion bioreactor for use with compliant tissue engineering scaffolds. *J. Biotechnol.* **133**, 490–496.
129. Yamamoto, H., Kim, E.J., Cho, S.W., and Jung, H.S. (2003) Analysis of tooth formation by reaggregated dental mesenchyme from mouse embryo. *J. Electron Microsc.* **52**, 559–566.
130. Ye, L., Mishina, Y., Chen, D., Huang, H., Dallas, S.L., Dallas, M.R., Sivakumar, P., Kunieda, T., Tsutsui, T.W., Boskey, A., Bonewald, L.F., and Feng, J.Q. (2005) Dmp1-deficient mice display severe defects in cartilage formation responsible for a chondrodysplasia-like phenotype. *J. Biol. Chem.* **280**, 6197–6203.
131. Honda, M.J., Sumita, Y., Kagami, H., and Ueda, M. (2005) Histological and immunohistochemical studies of tissue engineered odontogenesis. *Arch. Histol. Cytol.* **68**, 89–101.

132. Young, C.S., Kim, S.W., Qin, C., Baba, O., Butler, W.T., Taylor, R.R., Bartlett, J.D., Vacanti, J.P., and Yelick, P.C. (2005) Developmental analysis and computer modelling of bioengineered teeth. *Arch. Oral Biol.* **50**, 259–265.
133. Chung, I.H., Choung, P.H., Ryu, H.J., Kang, Y.H., Choung, H.W., Chung, J.H., and Choung, Y.H. (2007) Regulating the role of bone morphogenetic protein 4 in tooth bioengneering. *J. Oral Maxillofac. Surg.* **65,** 501–507**.**
134. Honda, M.J., Shinohara, Y., Sumita, Y., Tonomura, A., Kagami, H., and Ueda, M. (2006) Shear stress facilitates tissue-engineered odontogenesis. *Bone* **39**, 125–133.
135. Hu, B., Nadiri, A., Kuchler-Bopp, S., Perrin-Schmitt, F., Peters, H., and Lesot, H. (2006) Tissue engineering of tooth crown, root, and periodontium. *Tissue Eng.* **12,** 2069–2075.
136. Arany, S., Kawagoe, M., and Sugiyama, T. (2009) Application of spontaneously immortalized odontoblast cells in tooth regeneration. *Biochem. Biophys. Res. Commun.* **381**, 84–89.
137. Bhatt, A. and Le, A.D. (2009) Craniofacial tissue regeneration: Where are we?. *CDA J*. **37**, 799–803.
